# The role of E-learning in institutions of higher education in achieving the goals of sustainable development in Jordan

**DOI:** 10.1371/journal.pone.0319192

**Published:** 2025-03-21

**Authors:** Areej Derbas, Hani Y. Ayyoub, Tasneem Hyarat, Adnan Hnaif, Raed Al-Quraan, Amani Al-Serhan, Marwan Al-Tawil, Aida Al-Awamleh, Walaa Quteshat, Ismail Al Zyoud

**Affiliations:** 1 Department of Sociology, University of Jordan, School of Art, Amman, Jordan; 2 University of Jordan, King Abdullah II School of Information Technology, Development and Management Office for E-Learning Platforms, Amman, Jordan; 3 Department of Geology, University of Jordan, School of Science, Amman, Jordan; 4 Department of Cyber Security, Al-Zaytoonah University of Jordan, Faculty of Science and Information Technology, Amman, Jordan; 5 University of Jordan, King Abdullah II School of Information Technology, Amman, Jordan; 6 University of Jordan, Center for Women’s Studies, Amman, Jordan; 7 Department of Computer Information Systems, University of Jordan, King Abdullah II School of Information Technology, Amman, Jordan; 8 Department of Instruction and Supervision, University of Jordan, School of Physical Education, Amman, Jordan; University of Coimbra: Universidade de Coimbra, PORTUGAL

## Abstract

E-learning plays an important role in achieving the *Sustainable Development Goals* (*SDGs*). This research aimed to E-Learning’s impact on attaining SDGs in Jordanian Higher Education with a primary focus on the University of Jordan as a case study. The study was conducted on a sample of 3,000 students at the University of Jordan from various majors and academic levels and for both genders. The study adopted the quantitative statistical analysis method where a questionnaire was distributed electronically to students through the official platforms approved by the university. The results of the research showed that there is a positive role for E-learning in Higher Education institutions in achieving the sustainable development goals in Jordan, especially SDGs (1, 2, 4, 5, 7, 8, 9, 11, 12, 15, 16 and 17). Through the university’s efforts to develop the skills of students and faculty members in the field of technology and innovation, and holding seminars and conferences via E-learning platforms that enable universities to disseminate valuable information, participate in open dialogues, and raise awareness about SDGs and how to achieve them. Despite these efforts, more remain required to work towards the achievement of SDGs (3, 6, 10, 13, and 14).

## 1. Introduction

Over the past years, the world has focused greatly on the importance of sustainable development in a world facing many challenges. The idea of sustainable development has changed over the years and has come to represent more than its original purpose of protecting the environment with the gradual integration of economic development as well as social justice. The essence of the term sustainable development as expressed in the triple bottom line is that all issues must be addressed within the same time frame if adequate development is to be achieved [1].

The Framework further highlights the sustainable development Agenda enshrined in the United Nations’ 2030 Agenda for Sustainable Development which contains 17 Goals that are Better for the World. This strategy addresses the complexity of global issues and offers guidance concerning environmental, social, and economic aspects[2].

The 17 SDGs target the interrelated five pillars of the people-first approach to and implementation of the 2030 Agenda: people, planet, prosperity, peace, and partnerships. These five points elucidate that SDGs are not to be viewed in isolation because each of them is complementary to the other.[3]. People are those who are highlighted and it is the first pillar, “People-P1” whose goals include the following End poverty in all its forms everywhere (regulated 1), End hunger, achieve food security and improved nutrition, and promote sustainable agriculture, good health and wellbeing (SDG-2), Quality Education (SDG-4), Gender Equality (SDG-5). The second pillar, ‘Planet P2’ consists of goals such as Clean Water and Sanitation (SDG-6), Sustainable cities and communities (SDG-11), Sustainable consumption and production patterns (SDG-12), Climate Action (SDG-13), Life below water (SDG-14), Life on land (SDG-15). In light of the third pillar as a result of prosperity “prosperity (P-3)” includes objectives of Clean and affordable energy (SDG-7) decent work and economic growth Jobs (SDG-8), Industry, Innovation, and Infrastructure (SDG-9), and Reduced inequalities SDG 10. In the fourth pillar “Peace -P4” the goal is peace and justice (SDG-16). Finally, the fifth piece, ‘Partnership –P5’ focuses provision of partnerships covering the goals (SDG-17). Given the fact, that these five pillars are worked into policy and action, nations can work towards the SDGs and thus the healthy environment to which every individual is entitled.

The present reality makes it apparent why sustainable development is paramount from a Global perspective. Climate change, resource depletion, and loss of biodiversity are no longer hypothetical projections but are challenges that affect people today. The IPCC has been cautioning for years now that if countries do not take urgent action today to cut back on greenhouse gas emissions, the consequences are dark. In like manner, the alarming erosion of biodiversity is also said to affect food systems and the well-being of humanity. [4]

Therefore, as different countries take steps towards the achievement of SDGs, the contribution of education in general and higher education in particular has been emphasized as an essential component for the success of sustainable development. In this sense, higher education institutions are responsible for training future leaders, creators, and decision-makers who will be further engaged in achieving the goals set in the SDGs. As such, e-learning has now been recognized as an effective method for promoting the sustainable development agenda. E-learning, which is defined as the use of information and communications technology to deliver a course or program of study away from the conventional environment [5], also comes with great advantages that are geared towards the following sustainable development objectives:

1. Improving the provision of appropriate education (SDG 4) as it facilitates/internationalizes the provision of education regardless of the location or time.
2. The promotion of equity - potentially reducing gaps (SDG10) in educational opportunities
3. A decrease in the need for resources and travel linked to the education system model, which is based on a more than often irrational consumption pattern in order to support pedagogical aims by promoting more responsible consumption behaviors on behalf of the participants.

The importance of e-learning in furthering the cause of sustainable development has received additional emphasis with global phenomena such as the COVID-19 pandemic, which hastened growth in the usage of e-learning approaches by stakeholders around the world. This expansion occurred at a fast pace but was associated with both the benefits and problems of e-learning in ensuring educational accessibility and encouraging sustainable measures. The use of e-learning in higher education institutions in Jordan, which constitutes an important sector, may enable the country to achieve sustainable development goals through the effective use of e-learning strategies in achieving these goals. [6]. The aim of the use of e-learning in higher education in Jordan can use effective approaches to solve important problems of sustainable development:

1. Contribute to water conservation efforts (SDG 6) by raising awareness and providing specialized training on water management techniques.
2. Move towards renewable energy (SDG 7) by facilitating knowledge transfer and skills development in this rapidly evolving field.
3. Enabling higher education institutions to benefit from global knowledge networks and resources, thus enhancing the quality of higher education and aligning educational programs with the skills needed for sustainable development.

However, the successful implementation of e-learning for sustainable development in higher education institutions in Jordan faces several challenges, including issues related to faculty training, student readiness, and the need for culturally and contextually relevant e-learning content. Addressing these challenges requires a concerted effort from policymakers, educational institutions, and other stakeholders. [7]. E-learning plays a crucial role in advancing the Sustainable Development Goals by expanding access to education, raising awareness, and facilitating knowledge sharing on a global scale [8]. It is imperative to harness the power of e-learning and technology and address the challenges associated with it to create a more sustainable, just, and prosperous future for all. Therefore, this research came to identify the role of e-learning in institutions of higher education in achieving the goals of sustainable development in Jordan by using quantitative statistical analysis. This study was conducted on a sample of students from the University of Jordan from various majors and academic levels and for both genders.

To address the main question of our research—“Does e-learning in higher education institutions help achieve the Sustainable Development Goals?”—the following research questions were established:

1. How does e-learning support SDGs relate to people (e.g., education, health, and equality)?
2. In what ways does e-learning contribute to environmental sustainability (planet)?
3. How does e-learning influence economic growth and innovation (prosperity)?
4. What role does e-learning play in promoting peace and stability?
5. How does e-learning foster partnerships for sustainable development?

## 2. Literature review

In this section, we review highlights e-learning’s role in achieving Sustainable Development Goals (SDGs) across areas like People, Planet, Prosperity, Peace, and Partnerships. It emphasizes how e-learning improves education access, promotes skill development, supports poverty reduction, gender equality, and environmental sustainability, and fosters global collaboration, contributing to economic growth and innovation.

### 2.1. The role of E-learning in achieving development goals

This section can be grouped into 5 categories:

#### 2.1.1. *People (P1).*

E-learning digital platforms serve as a transformative force in advancing various Sustainable Development Goals (SDGs). These platforms enhance access to educational content and promote economic development. By facilitating skill acquisition and boosting employability, e-learning plays a crucial role in addressing poverty-related challenges [9]. Furthermore, according to [10], E-learning platforms have a crucial role in enhancing economic development and entrepreneurship developing regions through resources that empower management and business skills.

E-learning contributes to ending hunger by providing agricultural training, nutrition education, and knowledge sharing [11]. Leveraging digital technologies enhances agricultural practices, improves food security, and supports sustainable food systems. The World Food Programme (WFP) utilizes e-learning to deliver targeted training on food security and sustainable farming practices to vulnerable communities, creating a more inclusive and sustainable food system[12, 13]. Through access to agricultural training and knowledge, online courses contribute to improving farming techniques, crop management, and soil conservation, ultimately reducing hunger and malnutrition[14].

Additionally, e-learning enhances health outcomes by expanding access to healthcare training and health literacy, while also spreading crucial health information to empower individuals to take charge of their well-being [15,16]. Furthermore, e-learning enhances quality education by promoting personalized learning for learners with different backgrounds [17]. It can also support gender equality by improving digital skills for girls and women [18]. This calls to having collaborative efforts to ensure equitable access to technologies [19].

#### 2.1.2. *Planet (P2).*

United Nations has acknowledged the key role of e-learning in promoting water management and increasing climate change awareness and other environmental issues, by providing training courses and accessible educational content [20]. According to [21], awareness of water conservation and use could be enhanced through e-learning online platforms. Furthermore, e-learning platforms advance sustainable practices worldwide by providing important information and skills [22]. In order to sustain water consumption and production, e-learning platforms provide training on sustainable production methods, enabling individuals to make better decisions and share best practices [23,24]. Furthermore, e-learning platforms encourage collaboration to enhance understanding and share knowledge that is needed to address climate change on a global scale [25]. Regarding marine conservation, e-learning platforms can provide valuable content and collaboration features to share knowledge about the importance of oceans and marine resources [26]. It can also offer global access to increase awareness of environmental such as sustainable agriculture and renewable energy [27,28]. The work by [29] and [30] showed that e-learning is important for improving the quality of life by helping cities and communities become more inclusive, safe, resilient, and sustainable. It allows people to share knowledge and build skills, which helps communities solve urban challenges like improving infrastructure and protecting the environment.

#### 2.1.3. *Prosperity (P3).*

Ensuring the availability of affordable, reliable, environmentally friendly, and modern energy for all, e-learning serves a critical function by increasing awareness, supporting the distribution of knowledge, and improving skills in renewable energy and energy efficiency. It does so by providing essential information on sustainable energy sources, encouraging efficient knowledge sharing, and strengthening collaboration across the energy sector [31, 32]. Furthermore, e-learning contributes to economic progress, widespread employment, and quality work by delivering skill development, supporting entrepreneurial ventures, and expanding access to job opportunities. It helps individuals gain relevant job skills, stimulates entrepreneurship, and connects them with remote work opportunities and global employment platforms, thus boosting economic growth. [33, 34].

In addition, e-learning is vital in strengthening infrastructure, driving inclusive and sustainable industrial growth, and nurturing innovation by providing access to technical expertise, facilitating technology exchange, and promoting collaboration. It helps individuals acquire technical competencies, encourages creativity, and connects people working on sustainable projects. [33]. E-learning plays a crucial role in reducing inequality within and among countries by leveraging digital technologies to increase access to education, promote digital inclusion, and foster knowledge sharing [35, 36]. It achieves this goal by providing flexible learning options through online courses and virtual classrooms, allowing individuals worldwide, regardless of their location or socioeconomic background, to access quality education and acquire relevant skills [37, 38]. Additionally, e-learning addresses the digital divide by ensuring accessibility on various devices, including mobile phones, making educational content available to those with limited access to traditional institutions [39].

Efforts are also made to provide digital literacy training, ensuring widespread participation in e-learning initiatives. Furthermore, e-learning facilitates knowledge sharing and collaboration through online platforms, discussion forums, and collaborative tools, connecting learners, educators, and experts globally. This cross-cultural exchange contributes to breaking down barriers and fostering understanding among diverse populations [40, 41].

#### 2.1.4 *Peace (P4).*

E-learning contributes to achieving peace, justice, and strong institutions by promoting legal education, enhancing human rights understanding, and strengthening the rule of law. Leveraging digital technologies, it provides access to legal courses and training for individuals, including aspiring lawyers and justice sector professionals [42]. E-learning resources offer comprehensive legal knowledge, case studies, and practical skills training, fostering a better understanding of legal systems and principles. Additionally, it raises awareness of human rights and social justice through online courses, promoting equality and justice. Interactive modules engage learners in critical thinking, contributing to a more informed and empathetic society [43].

E-learning platforms also strengthen the rule of law by promoting transparency, accountability, and access to legal information, empowering citizens to make informed decisions and enhancing trust in the legal system [44, 45].

#### 2.1.5 *Partnerships (P5).*

E-learning significantly contributes to the Global Partnership for Sustainable Development by fostering collaboration, knowledge sharing, and capacity building among diverse stakeholders. Enabled by digital technologies and online platforms, it plays a crucial role in promoting global collaboration and networking [46]. E-learning platforms provide virtual spaces for individuals, organizations, and institutions worldwide to connect, exchange ideas, and share best practices, thereby strengthening partnerships and fostering cross-cultural dialogue.

These resources offer opportunities for stakeholders to acquire knowledge and skills related to environmental conservation, climate change mitigation, and social equity [47] While leveraging digital technologies, it is essential for e-learning initiatives to be culturally sensitive, considering diverse perspectives and local contexts to ensure their relevance and effectiveness in achieving the goals of sustainable development.

## 3. Methodology

This research employed quantitative methods, administering a questionnaire to participants. The questionnaire was prepared and distributed to students enrolled in the Ethics and Human Values module at the University of Jordan. This module was selected because it includes students from various disciplines, and its content addresses topics relevant to the research focus [48]. The distribution and data collection were precise and non-repetitive, as each student had a unique username through the Moodle e-learning system, allowing them to complete the questionnaire electronically via a Google link. The questionnaire was distributed during the second semester of the academic year 2022/2023 [49].

The accuracy of data collection was ensured by allowing students to complete the questionnaire only once, facilitated by their unique login credentials. This process took place in the second semester of the 2022/2023 academic year. A random sampling technique was employed, and informed consent was obtained from all participants before data collection began [50]. No references to participants’ real identities are made in this research, as their privacy is fully protected [51].

The research adhered strictly to the ethical guidelines set by the University of Jordan, ensuring compliance with principles related to research involving human participants [52]. The study design and methodology were reviewed by a panel of experts to ensure ethical soundness, particularly regarding participant privacy, anonymity, and informed consent. Recommendations from the panel were implemented to align the research with high ethical standards.

Participants were assured of their anonymity through a survey introduction that explained the confidentiality of their personal information and the exclusion of identifiable data. Ethical guidelines recommended by the review panel and the University of Jordan were followed, ensuring data would remain confidential and securely deleted after the study’s publication [53].

**Fig 1:**
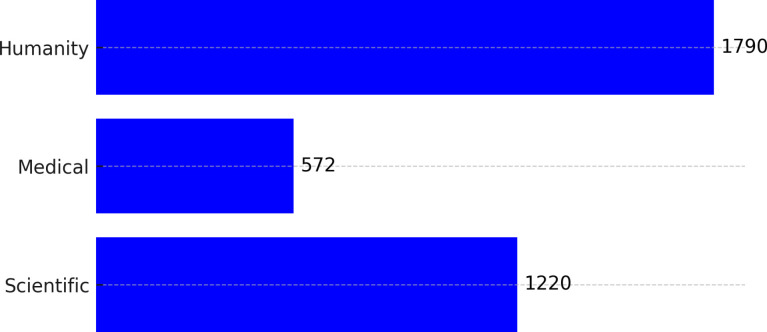
Number of participants according to faculties: Medical, Scientific and Humanities.

The questionnaire contained 24 items organized into five sections. The first section included six items related to “people,” the second eight items focused on “planet,” the third six items concerning “prosperity,” the fourth two items about “peace,” and the final section included two items related to “partnerships” [54]. A five-point Likert scale was used, ranging from strongly agree (5) to strongly disagree (1). Data from 3,000 participants were analyzed using the Statistical Package for the Social Sciences (SPSS), with 1,986 participants being female (66.2%) and 1,014 males (33.8%). Most participants were aged between 18 and 24, and 95.8% were first-year students. This study utilized Pallant’s[55].recommended methods for statistical analysis to process the data, and the detailed results are presented in Table 1 and Fig 1

**Table 1:** Characteristics of participants in the study.

		Frequency	Percent
Gender	Female	1986	66.2
	Male	1014	33.8
Age	≥ 25	126	4.2
	18-24	2874	95.8
Academic Year	First year	2318	77.3
	Second year	448	14.9
	Third year	76	2.5
	Fourth year	158	5.3
	Total	3000	100

The reliability of the questionnaire was assessed using Cronbach’s alpha, with a random sample of 582 participants selected from the total sample for the test. The results indicated a high reliability (alpha =  0.95), as shown in Table 2, which suggests that the questionnaire is reliable and suitable for use in the study [56]. The reliability of the five sections of the questionnaire, as presented in Table 3, ranged between 0.77 and 0.95, further supporting the instrument’s high degree of reliability. According to [57] guidelines, a reliability coefficient of 0.70 is considered the minimum acceptable threshold, reinforcing the reliability of the questionnaire for field application [58].

**Table 2:** Reliability test for Test Sample (N = 582).

Cronbach’s Alpha	Cronbach’s Alpha Based on Standardized Items	N of Items
0.95	0.95	582

**Table 3:** Reliability test for the five sections.

Sections	Number of Questions	Reliability
1	6	0.85
2	6	0.83
3	8	0.88
4	2	0.79
5	2	0.77
+	24	0.95

A descriptive analysis was conducted for each of the five sections, employing statistical measures such as the total number of observations, mean, and standard deviation. The methodology for conducting this analysis was guided by the techniques outlined by Field [54],and the results are presented in Tables 4–8.

**Table 4:** Descriptive statistics for (P1) People.

Questions		Strongly Agree	Agree	Neutral	Disagree	Strongly Disagree	Mean	Std. Deviation
(1) E learning plays a role in increasing the number of learners.	N	521	1601	540	179	159	3.72	0.994
	%	17.4	53.4	18.0	6.0	5.3		
(2) E learning has a role in improving the quality of education through the ability to utilize modern E learning techniques.	N	547	1587	516	206	144	3.73	0.994
	%	18.2	52.9	17.2	6.9	4.8		
(3) E learning plays a role in increasing health awareness and promoting health education	N	468	1489	630	264	149	3.62	1.0
	%	15.6	49.6	21.0	8.8	5.0		
(4) E learning contributes to the availability of Open Educational Resources (OER).	N	591	1589	559	135	126	3.79	0.948
	%	19.7	53.0	18.6	4.5	4.2		
(5) E learning plays a role in ensuring the continuity of education in exceptional circumstances.	N	884	1492	419	101	104	3.98	0.939
	%	29.5	49.7	14.0	3.4	3.5		
(6) E learning plays a role in empowering women in society.	N	523	1412	690	231	144	3.65	1.0
	%	17.4	47.1	23.0	7.7	4.8		

**Table 5:** Descriptive statistics for (P2) Planet.

Questions		Strongly Agree	Agree	Neutral	Disagree	Strongly Disagree	Mean	Std. Deviation
(1) E learning plays a role in promoting awareness to access clean water resources.	N	381	1436	740	284	159	3.53	1.0
	%	12.7	47.9	24.7	9.5	5.3		
(2) E learning contributes to increasing awareness of how to preserve our surrounding environment.	N	442	1580	633	216	129	3.66	0.959
	%	14.7	52.7	21.1	7.2	4.3		
(3) Good E learning contributes to increasing awareness of the mechanisms for reducing environmental pollution.	N	436	1601	645	200	118	3.68	0.937
	%	14.5	53.4	21.5	6.7	3.9		
(4) E learning reduces the need for mobility, thus helping to decrease environmental pollution and traffic congestion.	N	577	1540	583	177	123	3.76	0.966
	%	19.2	51.3	19.4	5.9	4.1		
(5) The use of modern technological means helps to reduce the consumption of educational materials such as paper and ink.	N	539	1584	566	184	127	3.74	0.963
	%	18.0	52.8	18.9	6.1	4.2		
(6) The change in learner behavior through E learning has led to increased opportunities for adapting to climate change (such as global warming, water scarcity, etc.).	N	424	1417	759	249	151	3.57	0.998
	%	14.1	47.2	25.3	8.3	5.0		
(7) E learning contributes to increasing awareness of the importance of marine life and the need for its protection.	N	377	1497	755	238	133	3.58	0.959
	%	12.6	49.9	25.2	7.9	4.4		
(8) Electronic learning platforms such as Zoom, MS Teams, and Facebook Live contribute to increasing awareness about environmental issues such as desertification through seminars, courses, and conferences.	N	429	1578	666	190	137	3.65	0.955
	%	14.3	52.6	22.2	6.3	4.6		

**Table 6:** Descriptive statistics for (P3) Prosperity.

Questions		Strongly Agree	Agree	Neutral	Disagree	Strongly Disagree	Mean	Std. Deviation
(1) E learning plays a role in emphasizing the importance of utilizing alternative energy and promoting a shift in individuals’ lifestyles to conserve energy. This is achieved by enhancing public awareness in this regard.	N	442	1528	687	198	145	3.64	0.973
	%	14.7	50.9	22.9	6.6	4.8		
(2) E learning enhances the technological skills that are considered future employability skills.	N	496	1598	577	211	118	3.71	0.955
	%	16.5	53.3	19.2	7.0	3.9		
(3) E learning provides opportunities for enhancing human development and enables the utilization of technology in planning and developing societal infrastructure.	N	455	1683	587	164	111	3.74	0.911
	%	15.2	56.1	19.6	5.5	3.7		
(4) E learning reduces the social gap within society.	N	397	1384	736	303	180	3.51	1.0
	%	13.2	46.1	24.5	10.1	6.0		
(5) E learning reduces the economic gap within society.	N	384	1394	774	287	161	3.52	1.0
	%	12.8	46.5	25.8	9.6	5.4		
(6) E learning reduces the educational gap within society.	N	472	1462	648	252	166	3.61	1.0

**Table 7:** Descriptive statistics for (P4) Peace.

Questions		Strongly Agree	Agree	Neutral	Disagree	Strongly Disagree	Mean	Std. Deviation
(1) E learning contributes to raising awareness to confront extremism and societal violence through seminars and conferences that use E learning tools (zoom, MS teams, Facebook live).	N	497	1635	577	164	127	3.74	0.943
	%	16.6	54.5	19.2	5.5	4.2		
(2) E learning contributes to the promotion of justice, peace and sustainable development by providing fair and equal educational opportunities.	N	441	1555	642	236	126	3.65	0.965
	%	14.7	51.8	21.4	7.9	4.2		

**Table 8:** Descriptive statistics for (P5) Partnership.

Questions		Strongly Agree	Agree	Neutral	Disagree	Strongly Disagree	Mean	Std. Deviation
(1) E-learning allows for partnerships with local community institutions.	N	411	1659	632	155	143	3.68	0.939
	%	13.7	55.3	21.1	5.2	4.8		
(2) E-learning enables global partnerships in the field of education.	N	543	1704	507	132	114	3.81	0.912
	%	18.1	56.8	16.9	4.4	3.8		

## 4. Results

This section presents the study’s findings, aiming to uncover whether e-learning in Higher Education institutions contributes to achieving the SDGs. The results address the research questions as follows:

### 4.1. E-learning’s Role in Supporting People-Centered Sustainable Development Goals

Table 4 shows the responses of the participants on the first pillar (P1) “People”. It showed that e-learning plays a role in achieving SDGs 1, 2, 4, and 5.

### 4.2. The Contribution of E-learning to Environmental Sustainability

Table 5 shows the responses of the participants on the second pillar (P2) “Planet”. It showed that e-learning plays a role in achieving SDGs 11, 12, and 15.

### 4.3. E-learning’s Impact on Economic Growth and Innovation

Table 6 shows the responses of the participants on the third pillar (P3) “Prosperity”. It showed that e-learning plays a role in achieving SDGs 7, 8, and 9.

### 4.4. The Role of E-learning in Promoting Peace and Stability

Table 7 shows the responses of the participants on the fourth pillar (P4) “Peace”. It showed that e- learning plays a role in achieving SDG 16.

### 4.5. Fostering partnerships for sustainable development through E-learning

Table 8, shows the responses of the participants on the fifth pillar (P5) “Partnership” showed that e-learning plays a role in achieving SDG17.

## 5. Discussion

This section presents the study’s discussion, aiming to uncover whether e-learning in Higher Education institutions contributes to achieving the Sustainable Development Goals (SDGs). The discussion of results as follows:

### 5.1. E-learning’s Role in Supporting People-Centered Sustainable Development Goals

Table 4 shows the responses of the participants on the first pillar (P1) “People”. It showed that e-learning plays a role in achieving SDGs 1, 2, 4, and 5. By expanding the use of e-learning and offering free educational content, we can make significant strides in eliminating poverty (SDG 1) [20], thereby reducing poverty rates and broadening educational opportunities for disadvantaged groups. Additionally, these findings underscore the pivotal role that the University of Jordan plays in realizing the objective of ending hunger and promoting food security (SDG 2). By empowering students and researchers and equipping them with knowledge and skills, they can actively contribute to innovative solutions that enhance food production and distribution, fostering food security both nationally and regionally [12, 13].

In the context of (SDG 4), which centers on enhancing education quality and accessibility, the University of Jordan’s efforts in leveraging advanced educational technologies and data analysis are vital [17]. In addition, e-learning is a key driver in advancing gender equality (SDG 5) by ensuring equal access to higher education and educational resources [18,59]. Whereas the lowest response among the sample individuals was to SDG 3, which represents the role of e-learning in increasing health awareness and promoting health education. This result can be attributed to the scarcity of interactive platforms aimed at increasing health awareness and education within the community, despite the community’s ability to access them, contributes to this outcome.

### 5.2. The Contribution of E-learning to Environmental Sustainability

Table 5 shows the responses of the participants on the second pillar (P2) “Planet”. It showed that e-learning plays a role in achieving SDGs 11, 12, and 15 through a multifaceted approach, where the University of Jordan plays a pivotal role through conducting crucial environmental research in many disciplines such as climate change, water resources, energy, and wastewater treatment. Moreover, the university champions sustainable practices by transforming the campus into environmentally friendly spaces. This involves implementing green initiatives like waste reduction, and energy efficiency. Additionally, the University of Jordan invests in and explores renewable energy sources, contributing to a cleaner and greener energy landscape. It has also begun using solar energy for electricity generation.

Through educational programs, they integrate environmental sustainability across various academic disciplines and organize awareness campaigns to educate both students and communities on environmental issues. Actively participating in community engagement, the University of Jordan collaborates with local partners on environmental projects and advocates for sustainable policies. Additionally, these results show that he lowest response among the sample individuals was to SDGs 6, 13, and 14. This can be attributed to the lack of sufficient awareness and knowledge about water conditions, climate change, and marine life protection in Jordan.

### 5.3. E-learning’s impact on economic growth and innovation

Table 6 shows the responses of the participants on the third pillar (P3) “Prosperity”. It showed that e-learning plays a role in achieving SDGs 7, 8, and 9 through enhancing public awareness of the importance of using alternative energy through research projects conducted within the university [32]. Professors and students can collaborate on projects promoting sustainable energy and disseminate their findings and recommendations to the community.

Through online courses and workshops, the university can boost students’ and faculty members’ skills in technology and innovation [20,33]. This, in turn, contributes to preparing graduates for gainful employment and fosters economic growth. The university provides opportunities for e-learning and participation in international conferences online. This helps enhance human development and enables the utilization of technology in developing community infrastructure[21,31]. The university can offer online education opportunities, reducing social disparities by providing access to education for all [37].It also diminishes economic disparities by lowering learning costs and improving the quality of educational content for better learning outcomes [38].

The lowest response among the sample individuals was to SDG 10, which represents the reduction of e-learning’s impact on social inequality within the society. This result can be attributed to the fact that e-learning requires advanced and modern devices and tools that support e-learning programs, as well as a strong and costly internet connection, which might hinder some individuals from affording these expenses.

### 5.4. The Role of E-learning in Promoting Peace and Stability

Table 7 shows the responses of the participants on the fourth pillar (P4) “Peace”. It showed that e- learning plays a role in achieving SDG 16. E-learning serves as a powerful tool for promoting peace and sustainable development within Higher Education institutions, it’s contribute to raising awareness about confronting extremism and societal violence. Seminars and conferences conducted through e-learning platforms like Zoom, Microsoft Teams, and Facebook Live enable universities to disseminate valuable information and engage in open dialogues on topics related to peace, conflict resolution, and countering extremism.

These virtual events reach a broad and diverse audience, transcending geographical boundaries and making it easier for experts, researchers, and activists to share their insights, strategies, and experiences, ultimately contributing to peace-building efforts. Furthermore, e-learning supports the promotion of justice, peace, and sustainable development by providing fair and equal educational opportunities to a wider and more diverse range of students. The University of Jordan, for example, actively engages in this process by offering online courses and programs. These digital resources break down barriers related to location, accessibility, and flexibility, ensuring that educational opportunities are accessible to all, regardless of their background or circumstances.

In this way, the university plays a crucial role in promoting equality in education and supporting the principles of peace and sustainable development. The University of Jordan’s contribution to these efforts is exemplary, as it actively participates in e-learning initiatives that foster peace and equality in education. By hosting seminars, conferences, and online courses on relevant subjects, it facilitates dialogue and awareness on peace-related issues.

### 5.5. Fostering Partnerships for Sustainable Development Through E-learning

The University of Jordan plays a pivotal role in advancing sustainable development through its multifaceted initiatives and partnerships. Locally, the university actively engages with the community by offering tailored education and training programs, addressing the specific needs of the local population. On a global scale, the University of Jordan leverages its collaborations with international institutions and organizations, such as EuRopean Community Action Scheme for the Mobility of University Students (Erasmus+), AgroTechnology VET Centers to Train Future Farmers in Jordan and Palestine (AgroTec) [60], Boosting Innovative Solar Energy Technologies and Applications in Mediterranean Countries Education (INNOMED) [61], Improving Higher Education Quality in Jordan Using Mobile Technology for Integration of Disadvantaged Groups (MEQUITY) [62], and Trans-European Mobility Programme for University Studies (Tempus) projects. These partnerships facilitate academic exchange and cooperation, fostering a diverse and interconnected network of institutions committed to share sustainability goals.

Research and development are central to the university’s contribution to sustainable development. Its involvement in projects allows it to harness the potential of e-learning and collaborative research to develop sustainable solutions and innovative practices. Moreover, the University of Jordan actively participates in global learning initiatives like Enhancing ICT Competencies of Early Childhood Educators at HEIs in MENA Countries (ICT4EDU) [63] and Modernization of Teaching Methodologies in Higher Education: Eu Experience For Jordan And Palestinian Territory (METHODS) [64]. These initiatives focus on enhancing education and teaching methodologies and benefit from the university’s expertise, while promoting the exchange of knowledge and best practices on a global scale. As shown in Table 8, the responses of the participants on the fifth pillar (P5) “Partnership” showed that e-learning plays a role in achieving SDG17.

## 6. Conclusion

This study demonstrates the vital role of e-learning in higher education institutions, particularly in advancing the Sustainable Development Goals (SDGs) within the context of the University of Jordan. By leveraging e-learning platforms and fostering technological innovation, the university has effectively engaged its academic community in sustainable practices and global knowledge exchange. The use of tools such as Zoom, Microsoft Teams, and Facebook Live has been instrumental in disseminating information and encouraging meaningful discussions about sustainability.

The university’s efforts extend beyond e-learning platforms, encompassing participation in international initiatives like Erasmus + , Tempus, ICT4EDU, and METHODS. These collaborations have facilitated the adoption of innovative teaching methodologies and sustainable solutions. Additionally, the institution has made significant strides in integrating renewable energy sources and promoting research initiatives aimed at sustainability on campus.

The findings underscore the transformative potential of e-learning in fostering sustainable development within higher education, positioning the University of Jordan as a leader in achieving numerous SDGs. However, the study also highlights areas requiring further attention to ensure a more comprehensive impact on the remaining goals. These insights contribute to the broader discourse on sustainability, emphasizing the critical role of education and innovation in shaping a sustainable future.

## 7. Recommendations

To further enhance the role of e-learning in achieving the Sustainable Development Goals (SDGs), it is crucial to foster partnerships with local community institutions through collaborative e-learning initiatives that benefit both university students and the surrounding community. Increasing awareness of water conservation and access to clean water through online learning modules will address Jordan’s pressing water scarcity issues. Additionally, providing community education on health topics via online platforms can promote health literacy and improve public health outcomes. Developing e-learning programs that focus on social and economic equality will help participants understand and advocate for equitable practices within their communities. Finally, expanding e-learning courses that address climate change, including strategies for adaptation and mitigation, will enhance community resilience to environmental challenges.

## 8. Limitations

This study acknowledges several limitations. The sample was limited to students at the University of Jordan, which may not fully represent the experiences of students at other institutions or in different regions. Additionally, the reliance on self-reported data may introduce bias, as participants may respond in socially desirable ways rather than providing their true opinions.

## 9. Directions for future research

Future research could explore the impact of e-learning on achieving SDGs in various higher education contexts, including comparisons across different universities and regions. Longitudinal studies could assess how e-learning initiatives evolve over time and their sustained impact on SDG achievement. Additionally, qualitative research could provide deeper insights into students’ and faculty’s experiences with e-learning, allowing for a more nuanced understanding of its effectiveness in promoting sustainable development.
